# An Omitted Radiological finding in the Pediatric Age Group: Physiological Sacroiliac Joint Vacuum Normal Variant

**DOI:** 10.51894/001c.27361

**Published:** 2021-08-30

**Authors:** Emrah Doğan, Hüseyin Aydoğmuş, Sinem Aydoğmuş

**Affiliations:** 1 Radiology Muğla Sıtkı Koçman University; 2 Physical medicine and rehabilitation Muğla Education and Research Hospital

**Keywords:** sacroiliac joint, computed tomography, vacuum phenomenon, pediatric radiology

## Abstract

**INTRODUCTION:**

Gas accumulation in human joint spaces has been generally described as the vacuum phenomenon (VP). To date, the sacroiliac joint VP has been associated mostly with pathological, particularly degenerative conditions (e.g., arthritis, obesity, discal degenerations, fractures, dislocations, avascular necrosis).

**OBJECTIVE:**

The study aimed to examine the characteristics of the physiological form of VP and its radiological patterns in a sample of pediatric patients.

**METHODS:**

A sample of seventy patients between 0 and 17 years old (mean age, 11.4 ± 5.54) were included in the study. Sample VP cases was evaluated according to types, age group, anatomic localization, gender, and sides. RESULTS: Two (2.9%) of sample children had degenerative VP, with 24 (34.2%) of patients demonstrating physiological VP in the sacroiliac joints. VP rates significantly increased after nine years of age (p < 0.01) and 83% of physiological VP cases were determined to be bilateral.

**CONCLUSIONS:**

Although degenerative VP is a rare entity in children, non-pathological VP can be a more common aspect of sacroiliac anatomy. Although sacroiliac VP is frequently an underreported or omitted finding in imaging studies, this condition may be clinically important as a clue for other degenerative diagnoses. Normal variants of VP may be clinically important in children since they may mimic inflammatory and infectious pathologies during magnetic resonance imaging and computed tomography images.

## INTRODUCTION

Gas accumulation in the joint space has been generally described as the vacuum phenomenon (VP).^1^ To date, the sacroiliac joint (SIJ) VP has been most frequently associated with pathological, particularly degenerative conditions (e.g., arthritis, obesity, discal degenerations, fractures, dislocations, avascular necrosis).^2,3^ Physiological forms of VP in the pediatric age group have been presumed to be a rare phenomenon.^4,5^ As far as the authors could identify, articles in the English literature have been limited to normal variants of this condition.^5^ However, condition may be more clinically important in children since because it can mimic inflammatory and infectious pathologies on magnetic resonance (MRI) and computed tomography (CT) imaging.^6^

In 2016, Takata et al. evaluated the relationship between sacroiliac pelvic morphology and VP in all age groups.^7^ The only pediatric research in this subject was the study of You et al.^5^ that compared the SIJ VP and body mass index (BMI) in pediatric patients and their study is based on obesity and degenerative VP relationship.^5^ In another article from Lo et al., SIJ VP was classified in patients under 40 years old, 40-60 years old and over 60 years old without specifying pediatric patients.^4^ In summary, the published articles have not focused on the physiological pediatric SIJ VP. To the best of the authors’ knowledge, the pediatric SIJ VPs in terms of their physiological nature has been first evaluated during the project described in this article.

During musculoskeletal system evaluations of pediatric patients, MRI is often preferred instead of CT to avoid excess radiation risks. However, since gas is seen at different signal intensities on MRI, this preference may cause the VP to be confused mistakenly with pathological situations.^4,7^ There remains no clear terminology to distinguish uncaused VP from the pathological forms of VP, as this finding is rarely mentioned in the literature. Physiological VP, non-pathological VP are acceptable terminological descriptions. Physiological VP is the name used for this finding’s definition in a previous paper.^8^ As a result, the authors preferred to use this terminology in their study.

### Purpose of Study

Ultimately, the study aimed to describe the characteristics of the physiological VP and its radiological patterns in a sample of pediatric patients. The authors intended to systematically identify likely rates of VP in the SIJ and examine overall radiological characteristics of physiological forms of VP in the pediatric population.

## METHODS

Before data collection, this study was approved in 2020 by the authors’ university Human Research Ethics Committee. Eligible patients who had received CT scans between January 2016 and June 2020 for various indications were retrospectively evaluated using images obtained from accessible picture archiving communication systems (PACS). Sacroiliac joint VP was initially detected in a total sample of 79 pediatric patients. Five (6.3%) patients were excluded from analyses for the following medical reasons: one patient had a Wilms tumour, one had lymphoma, and three had a previous history of pertinent trauma. In addition, four CTs with artefacts were also excluded due to not being appropriate for evaluation.

In total, 70 children, male, 34 (48.5%) and female, 36 (51.4%); mean age, 11.4 ± 5.54) were eligible for study evaluation. The pre-study minimal sample size power calculations that had been conducted by the authors using G-power*3 software indicated that a necessary sample of 45 patients would be required to attain 95% power, 0.05 Alpha and 0.5 effect size in a one-way calculation (Critical t = 1.6802).

Sample children’s CT scans had each been performed with a 256-slice multi-detector CT scanner (Somatom, Siemens Healthcare, Erlangen, Germany). Sacroiliac joints were individually visualized in coronal, axial and sagittal planes. Oral sedatives were administered before the CT procedure [chloral hydrate is 50–100 mg/kg (up to a maximum of 2.0 g)]. The tube voltage (80 kV weight < 28 kg; higher voltages for 28.1–50.0 kg and > 50 kg as 100 kV and 120 kV) and applied radiation dose (2.3 mSv to 19.9 mSv) varied depending on the weight of each child. All CTs were conducted in a supine position. Other CT scan parameters were as follows: rotation time, 0.35s; thickness, 1mm; FOV (field of view) 30-40 cm.

**Figure 1.**
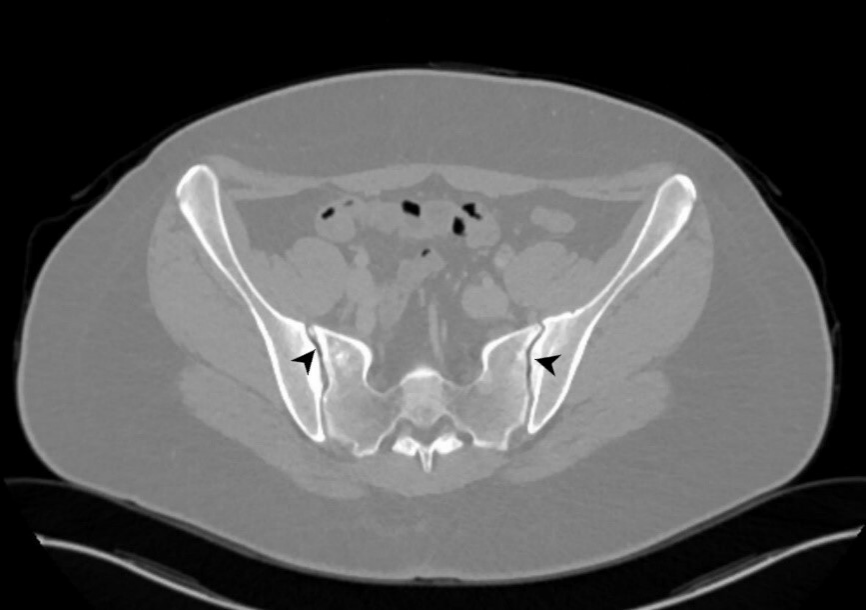
Bilateral air densities corresponding to VP in SIJs (arrows) on axial CT in the bone window.

Each study image was assessed by the two blinded radiologists (first author ED and third author SA). Each radiologist independently evaluated the CT images on the coronal, axial and sagittal planes. In case of contradictory results, the images were evaluated together by both radiologists. Imaging evaluations were made at the workstation with a high-resolution medical monitor in soft-tissue window [Windows width (WW): 400 Hounsfield unite (HU), windows level (WL): 50 HU], bone window (WW: 1800 HU, WL: 400 HU) as well as the lung window (WW: 1500 HU, WL: -600 HU) to distinguish air more clearly from soft tissue and bone. The presence of gas (i.e., air) was considered positive for VP in the SIJ (Figure 1).

**Figure 2.**
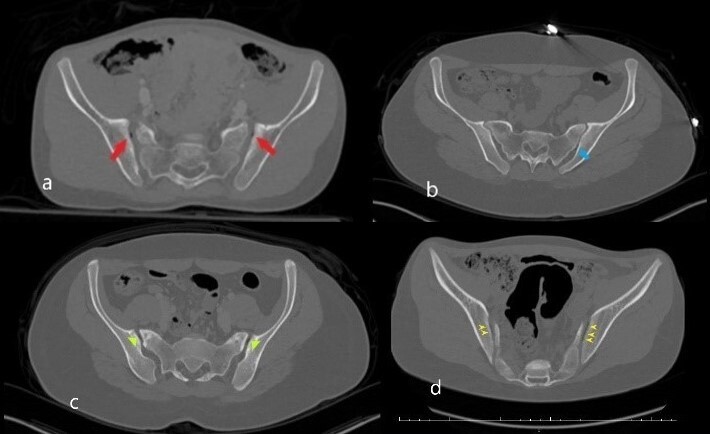
Bilateral point shaped VP with sclerosis in iliac surface and minimal irregularity (red arrows in a) b. Unilateral *VP* (blue arrow) located in the posterior part of the left SIJ c. Short linear shaped *VP* (green arrowhead) in bilateral anterior parts of the SIJs. Also, sclerosis in anterior sacral and iliac parts of the joint d. Bilateral long linear shaped *VP* (yellow arrowhead). The right side of the figure shows the degenerative VP (a and c) while the left side (b and d) the physiological ones.

Patient results were classified according to age groups (0-4 years old, 5-8 years old, 9-11 years old, 12-14 years old, 15-17 years old) as well as gender (male, female). Data according to the presence of SIJ VP were enlisted as right, left and bilateral (Figure 2).

The symmetry and asymmetry of the patterns were examined in patients with bilateral VP. If both sides were in the same patterns, it was accepted as symmetric, while asymmetric in different patterns.^9^ The patients were divided into VP and non-VP groups for comparison. If gas accumulation in the joint was detected, it was classified in the VP group regardless of whether it was unilateral or bilateral, otherwise, it was classified as non-VP. The related groups were compared according to the appropriate parameters and necessary analyses were completed.

Study data were stored using a Microsoft Office Excel spreadsheet (Excel 2010, Microsoft). The authors used software (SPSS, Version 22.0, IBM) for statistical analyses. Continuous variables were expressed as mean ± SD (standard deviation) values. Qualitative variables were totalled and calculated as percentages. All data were statistically compared according to gender. The Student’s t-test procedure was used for analysis of the mean of normally distributed values. Age groups were compared using the Student’s t-test and Analysis of Variance (ANOVA) test. Pearson chi-square (χ^2^) analysis was used to evaluate the relationship between variables. P values < 0.05 were statistically significant.

## RESULTS

This study was conducted with CT images of N = 70 patients. The mean age of male patients was 10.87 years ± 5.57; 0-17 years (age ± SD; age range). The mean age of female patients was 11.83 years ± 5.55 years; 0-17 years (age ± SD; age range). In total, the overall mean sample age was 11.4 years ± 5.54 years; 0-17 years (age ± SD; age range). Table 1 summarizes sample patient characteristics.

**Table 1.** The number and percentage of the <italic>physiological VP</italic> according to age groups, genders and sides.

**Position**	**Gender**	**0-4 years (n = 12)**		**5-8 years** **(n = 11)**		**9-11 years** **(n = 6)**		**12-14 years** **(n = 4)**		**15-17 years** **(n = 37)**	
**Unilateral** **VP**	Male	0	0%	0	0%	1	33.3%	0	0%	1	7.4%
	Female	0	0%	0	0%	0	0	0	0%	2	9.5%
**Bilateral** **VP**	Male	0	0%	0	0%	1	33.3%	2	100%	6	42.9%
	Female	0	0%	1	25%	2	66.7%	0	0%	8	38.1%
**Total**	Male	0	0%	0	0%	2	66.7%	2	100%	7	50%
	Female	0	0%	1	25%	2	66.7%	0	0%	10	47.6%

Two male patients, who are 15 years and 16 years old, both with obesity, had degenerative VP. Surface irregularity and sclerosis were present in their SIJ. VP was detected in 24 (48.6%) patients without any accompanying finding in favour of degeneration. These patients were included in the physiological VP subgroup. In total, 26 of 70 (37.1%) of the patients had VP. Two (2.9%) of 70 children were concluded to have degenerative VP, while 24 (34.2%) possessed physiological forms of VP.

Respectively the physiological VP distribution according to age groups as follows:

**0-4 years old:** None of the sample patients had VP.

**5-8 years old:** 9.1% (one of 11) of all patients and 25% (one of 4) of the female patients had VP while none of the male patients had VP in this age group. The VP was bilateral in this one female patient.

**9-11 years old:** 66.7% (4/6) of the children in this age group had VP. The percentages were equally distributed between both genders. 33.3% (1/3) in males had bilateral VP while 33.3% (1/3) of them unilateral. None of the female patients had unilateral VP in this age group. 66.7% (2/3) of the female patients had bilateral VP.

**12-14 years old:** Two (50%) of the patients in this age group had VP, both males.

**Figure 3.**
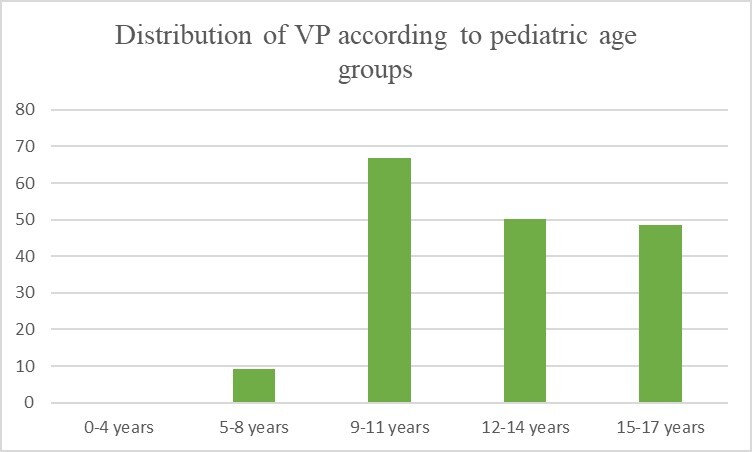
Percentages of VP patients by age groups.

**15-17 years old**: Two patients had degenerative VP in this age group. Totally, 45.9% (17 of 37) of the patients had SIJ VP. 43.8% (7 of 16) of male patients and 47.6% (10 of 21) of the female patients had VP. For male patients, 12.5% 2 of 16 had degenerative VP. In the evaluation of physiological VP, 7.4% (1 of 14) of the male patients had unilateral VP while 42.9% (6 of 14) of the had bilateral VP. 9.5% (2 of 21) of the female patients had unilateral VP while 38.1% (8 of 21) of the female patients had bilateral VP. (Figure 3)

Both patients with VP in each gender subgroups and the total sample were separately evaluated using the *ANOVA test*, with no significant correlations found between subgroups. However, each age group was consecutively compared with a higher age group using the Student’s *t*-test. There was a significant difference in evaluation between the 5-8 age group and the 9-11 age group in both genders (p < 0.01) .

Children who were under nine years of age and over nine years were stratified into two subgroups and compared with *Pearson chi-square* (χ^2^) analysis. P value was also highly significant (p < 0.01). In summary, there was an adequate imaging threshold for VP found when categorizing patients by this age parameter. However, “under and over nine” age group images were separately heterogeneous and did not regularly demonstrate VP increases. No statistically significant differences were found in the comparisons between 0-4 years and 5-8 years, between 9-11 and 12-14 years, and between 12-14 years and 15-17 years.

VP were compared across gender using Pearson’s chi-square (χ^2^) analysis. There were no overall statistically significant differences found between gender subgroups (p = 0.603) (Table 2).

**Table 2:** The gender distribution of <italic>physiological VP</italic> according to symmetry.

**Gender**	**Male n = 11**				**Female n = 13**				**P value**
**Symmetry**	**Symmetric**		**Asymmetric**		**Symmetric**		**Asymmetric**		
*Bilateral* *N = 20*	*7*	*63.6%*	*2*	*18.2%*	*5*	*38.4%*	*4*	*30.8%*	*Male/Female VP = 0.603* *Male/Female Symmetry < 0.05*
*Unilateral* *N = 4*	*-*	*0%*	*Total: 2* *Right: 1* *Left: 1*	*18.2%* *9.1%* *9.1%*	*-*	*-*	*Total: 4* *Right: 3* *Left: 1*	*30.8%* *23.1%* *7.7%*	
*Total*	*7*	*63.6%*	*4*	*36.4%*	*5*	*38.4%*	*8*	*61.6%*	

In the total sample evaluation of physiological VP performed regardless of parameter groups, VP was bilateral in 18 (75%) of 24 cases. The authors also evaluated pattern asymmetry in sample children who had bilateral VP. This pattern was symmetrical in 12 (66%) of 18 patients and asymmetrical in 6 (33%) of 18 patients. Six (25%) of 24 remaining children had unilateral VP, 4 (66.7) of six on the right side and 2 (33.5%) of six on the left side. There was a statistically significant difference between genders according to the symmetry of *SIJ VP* (p < 0.05).

**Figure 4.**
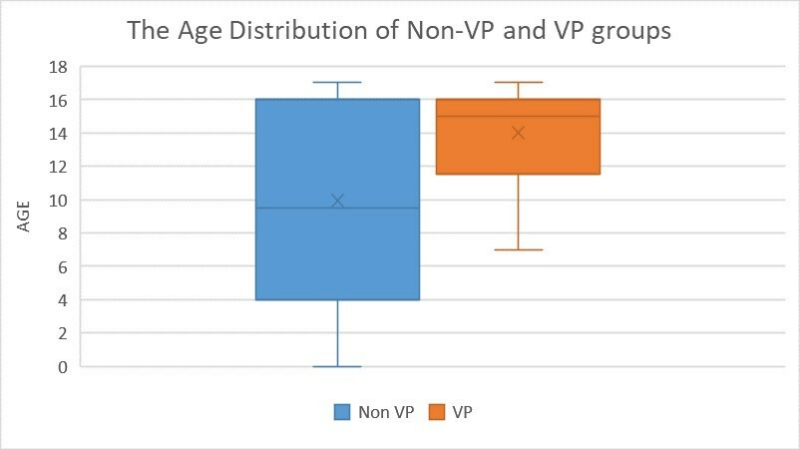
Age distributions of VP and non-VP and VP Subgroups.

The mean age of the non-VP group was 9.95 ± 6.05, while the VP group 14 ± 3.18. There was a statistically significant difference found between VP and non-VP age groups according to Pearson’s chi-square (χ^2^) test (p < 0.05) (Figure 4).

## DISCUSSION

VP in pediatric patients is a frequently neglected radiological finding. One of the reasons this condition is not often described in radiology reports is that it is generally considered unimportant. Radiologists are not inclined to dwell on describing results that cannot be used.^4,5^ However, diagnosis of paediatric VP may be helpful for more complex imaging scenarios. For example, VP can accompany arthritis, obesity, discal degenerations, fractures, dislocations and avascular necrosis.^10,11^

If clinicians encounter a fracture, the presence of VP may suggest an underlying chronic process.^12^ Also, intraarticular gas can be confused with the lucent crescent sign in case of avascular necrosis in X-ray.^13^ In addition, the air signal can mimic different appearances during MRI and may be misleading. This situation does not change depending on whether the VP is physiological or pathological.^10^ As a result, VP presence often influences diagnostic decisions, even if not emphasized during standard radiological reports.

**Figure 5.**
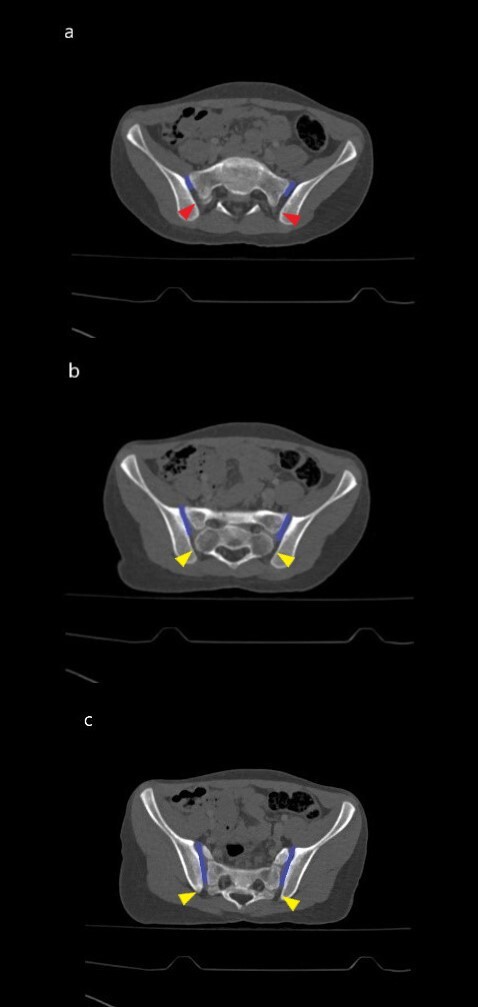
In axial CT images Anterior small part is synovial (blue line); the posterior big part is syndesmosis (red arrowhead) in the taken images of the upper part of joint b. In the midsection of the joint, the anterior half of the joint is synovial (blue line) whereas the posterior half is symphysis-like (yellow arrowhead) c. The anterior big part of the joint is synovial (blue line), only the small posterior part is symphysis-like in the lower part of the joint (yellow arrowhead).

The *SIJ* is the body’s largest joint.^14^ Roughly, the upper third of the joint is a syndesmosis (fibrous joint linked by a strong membrane or ligaments), the middle third *symphysis-like* and the lower third the *synovial*.^15^ However the synovial part is not limited to only the lower segment. It continues towards superior in an oblique course along the anterior part. In other words, most the superior part of SIJ is syndesmosis but a small part is synovial.^16^ This anatomical information is important for understanding and discussing the research on the right basis. Since the VP effect is in the synovial part, the gas is formed in this part of the joint Figure 5.^16–19^

As suggested by these results, VP is not always a stable condition. In fact, it is frequently a phenomenon of a pressure/solubility equilibrium. The presence or absence of VP may change according to different physical conditions in the radiological examinations performed at different times of childhood and adolescence.^20^ The VP doesn’t form if there is fluid in the joint.^21^ In 2017, Laloo et al. reported that VP findings were not observed in patients with arthritis.^22^ Juvenile arthritis is one of the important pathologies affecting SIJ.^23^ Laloo’s finding can also be used for diagnosis in pediatric radiological evaluation.^22^

VP will rarely induce symptoms. A condition called pneumatic nerve root compression associated with VP is a cause of pain.^24^ However, this entity is valid for the lumbar region. Apart from this, VP was associated with hip and back pains, but this symptom is attributed to pathological VP. To our knowledge, there is no data about symptomatology and physiological VP relationship in the literature.^5^ When VP is detected, it is important for clinicians to consider whether this finding is degenerative or physiological since SIJ degeneration is an important subsequent cause of low back pain.^25,26^

Pathological VP is the only condition confused in the differential diagnosis of physiological VP in CT imaging, as it can distinguish accompanied degenerative findings.^3,27,28^ Since CT is a technique with radiation, MRI is preferred in the pediatric age group. In MRI evaluation, loose body, amyloid, chondrocalcinosis (calcium deposition in cartilage), arthrofibrosis (a fibrotic joint disorder characterised by excessive collagen production) is in differential diagnosis with VP. ^29,30^ As seen here, this unreported finding by radiologists is also clinically important in the pediatric age group in many conditions.

### Study Limitations

Our study had some limitations. Our study conclusions have been drawn from a smaller retrospective convenience sample and limited to CT scan images. We acknowledge that the imaging and diagnostic resources available to clinicians may vary from our Turkish university setting.

## CONCLUSIONS

Physiological VP is one of the more common aspects of sacroiliac anatomy in the pediatric age group. Although SIJ VP is an underreported or omitted finding in imaging studies, this condition may be clinically important as a clue for other degenerative diagnoses. Additional studies with larger pediatric sample are required to further examine this condition. These results could be used for differential diagnosis from pathologies in MRI evaluation.

### Financial Disclosure

The authors declared that this case has received no financial support.

### Competing Interests

The authors have no competing interest.
